# Third‐generation posterior‐stabilised cemented system for total knee arthroplasty: Survivorship and outcomes after 20‐year follow‐up

**DOI:** 10.1002/jeo2.12063

**Published:** 2024-06-22

**Authors:** José Luis Patiño Contreras, Victoria Sebastián Pérez, Checa García Antonio, Montejo Sancho Jorge, Martínez Martín Javier

**Affiliations:** ^1^ Department of Orthopedic Surgery Hospital Universitario Fundación Alcorcón Alcorcón Madrid Spain

**Keywords:** knee arthroplasty, long‐term results, posterior‐stabilised, revision rate, survivorship

## Abstract

**Purpose:**

Total knee arthroplasty is a common procedure due to increased life expectancy and ageing populations, necessitating implants with long‐term efficacy. After some initial designs, the third‐generation modular posterior‐stabilised NexGen® prosthesis aimed to enhance kinematics and reduce complications. This study evaluates the long‐term outcomes, survivorship, revision rates and complications of this implant. With promising results observed up to 15 years in previous studies, this investigation aims to assess the implant's performance over extended follow‐up periods, aiding in optimal implant selection for improved patient outcomes.

**Methods:**

We carried out a retrospective study on 263 total knee arthroplasties performed in our centre between 1998 and 2002. Statistical analysis of complications was performed and study of survival using the Kaplan–Meier method and competing risk analysis were calculated. Description of reinterventions and complications were also included.

**Results:**

Results show a 20‐year prosthesis survival rate of 90.8% for revision due to any reason, with an estimated survival of 92.3% considering competitive events. Estimated survivorship at 20 years is 98% for aseptic loosening as the end point, and an estimation of 98.80% considering competitive events. Twenty revisions were performed, with 10 cases due to infection and 10 for noninfectious reasons and three of them due to aseptic loosening. Radiographic analysis revealed radiolucent lines, but no clinical evidence of loosening was observed in these cases.

**Conclusion:**

This study offers survivorship data from longer follow‐up periods, what is difficult to find in the reported literature and showed excellent results of this implant in terms of survivorship and low rates of revision in our cohort.

**Level of Evidence:**

Level IV.

**Abbreviations**
AP: anteroposterior
CI: confidence interval
CNS: staphylococcus coagulase negative
CR: cruciate retaining
KM: Kaplan–Meier
LCCK: legacy constrained condylar knee
LPS: legacy posterior‐stabilised
PS: posterior‐stabilised
RHK: rotating hinge knee
TKA: total knee arthroplasty

## INTRODUCTION

Total knee arthroplasty (TKA) is one of the most frequent surgeries performed in orthopaedic surgery units and the demand for this procedure is increasing as a consequence of the rise in life expectancy and the ageing of the population [15], which increases the need for implants with excellent long‐term results.

A satisfactory result of a TKA depends on restoring correct limb alignment, the correct placement of the implant and a correct balance in the soft tissues. These factors depend on the surgical technique and implant design, which have an impact on the longer durability of the arthroplasty [22].

After initial designs, a third‐generation modular posterior‐stabilised (PS) prosthesis was created in the mid‐1990s to improve kinematics, femoral tracking and to decrease the patellofemoral complications from previous designs, demonstrating promising results in short‐ and medium‐term studies [17].

One of these systems is the NexGen® (Zimmer) design, which was said to provide several advantages, such as a repositioned cam‐spine mechanism and a redesigned patellofemoral joint (lateralisation and a more extended and deeper femoral trochlear groove), in addition to a trochlear angle of 7°, closely resembling the natural angle of the knee.

Data collected from the long‐term studies of different TKA designs guide us in making the best decisions for our patients and thus selecting the optimal implant options.

The purpose of this study is to analyse the outcomes and survivorship during long follow‐ups, as well as the revision rates and complications for NexGen® (Zimmer) knee prostheses that were implanted in our centre between 1998 and 2002. This specific implant has shown excellent results in studies with up to 15 years follow‐up and results might be consistent after longer follow‐up periods.

## MATERIALS AND METHODS

This is a retrospective, observational, nonrandomized study carried out to analyse the survivorship and the revision rate of the posterior‐stabilised NexGen® (Zimmer) knee prosthesis that was implanted in our centre between 1998 and 2002. Only posterior‐stabilised TKA were included, and other levels of constraint were excluded from the study.

Institutional medical records were accessed to collect data and X‐rays of the patients, and 263 TKA met the inclusion criteria. In the cases where no recent data were found, patients were contacted by telephone to confirm the patient and prosthesis survival. Patients who could not be contacted or who were not followed at our institution were excluded. In six cases, it was not possible to contact the patients, who were considered lost to follow‐up and excluded from statistical analysis.

The 263 NexGen legacy posterior‐stabilised (LPS) included in the study were implanted in 221 patients: 178 females and 43 males. The average age at the time of surgery was 71.97 years (median: 72.7), ranging from 39 to 88 years.

### Surgical technique

Surgery was performed in all cases by a single team composed of two specialised arthroplasty surgeons, following the same treatment guidelines and surgical technique.

Surgery was carried out following placement of a pneumatic tourniquet and a 2 g dose of Cefonicid was used as antibiotic prophylaxis before surgery or 1 g of Vancomycin in those patients who were allergic to penicillin.

The medial parapatellar approach with resection of both cruciate ligaments as described by Insall has been the one used on every occasion. For tibial cuts, an extramedullary guide with a fixed 7° tibial slope was used. Femoral osteotomies were performed using an intramedullary cutting guide, with 5° of valgus for male patients and 7° for female patients, always at 3° of external rotation. All the prostheses have been cemented with CMW1 (DePuy) cement with gentamicin. No tranexamic acid or intraarticular anaesthetic infiltration has been used. No navigation system has been used. The patella was prosthetized in 15 patients, with the decision made at the discretion of each surgeon.

### Postoperative and follow‐up

In the postoperative period, all the patients started rehabilitation on the day after surgery, by means of physical therapy and continuous passive‐motion machine. Full weight‐bearing was allowed from the first postoperative day.

At the time of maximum follow‐up, the patients had an in‐person review at the clinic. For patients who had not been able to attend, survivorship of the prostheses was confirmed either by phone or by accessing recent medical records.

Serial anteroposterior (AP), lateral and patellar axial radiographs were reviewed to assess the presence and progression of osteolysis or loosening around the implant. Standing weight‐bearing AP and supine lateral X‐rays of the knees were carried out for every available patient at the point of maximum follow‐up. These X‐rays were evaluated by two independent surgeons, and a radiographic assessment of radiolucent lines was conducted according to the Modern Knee Society Radiographic Evaluation System [20].

### Statistical analysis

Statistical analysis was carried out using SPSS 25 (IBM) and STATA 17. This work got the Institution Review Boards' approval.

Survival time is defined from the primary surgery to one of the following: date of revision of any of the prosthetic components, until September 2021 if no revision surgery was performed, or the last known follow‐up or patient's decease.

The data were analysed using the Kaplan–Meier (KM) method and the survivorship function was estimated considering the revision surgery for any reason as the main event. A second KM curve was calculated, taking into consideration the revision due to aseptic loosening. Survival rates are shown at 10, 15 and 20 years, considering death as a censoring event.

The cumulative incidence of competing risk was estimated because the KM method can overestimate the risk for revision in the presence of competing risks, such as the death of the patient.

## RESULTS

### Survivorship

From the initial cohort, 103 patients had a follow‐up longer than 18 years and 56 cases longer than 20 years. At the time of the study, 76 patients were alive and found to still have their primary knee prosthesis (the average follow‐up of this group was 20.72 years).

**Figure 1:**
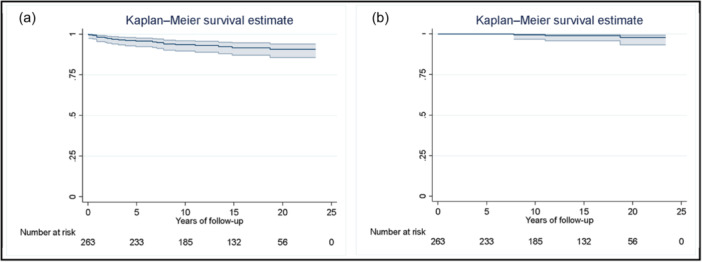
Kaplan–Meier survivorship curves with 95% confidence intervals (CIs) are shown: (a) revision for any reason (90.8% [95% CI: 85.7%–94.1%] at 20 years of follow‐up). (b) Revision due to aseptic loosening (98% [95% CI: 93.9%–99.4%] at 20 years of follow‐up) (colour printing needed).

**Figure 2:**
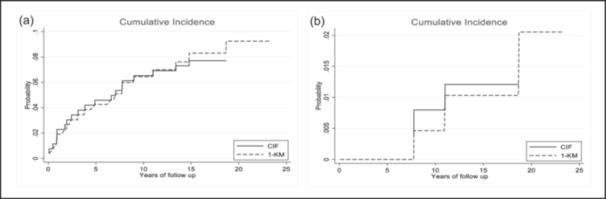
Cumulative incidence of revision in the presence of competing risks is shown. (a) Revision for any reason; (b) revision due to aseptic loosening. CIF, cumulative incidence function; KM, Kaplan–Meier.

The prosthesis survival rate at 20 years was 90.8% (95% confidence interval [CI]: 85.7%–94.1%) for revision due to any reason as the study event, and estimated survival of 92.3% in the presence of competitive events. The survivorship at 20 years is 98% (95% CI: 93.3%–99.4%) considering revision due to aseptic loosening as the end point, and an estimation of 98.80% survivorship in the presence of competitive events. Complete data are shown in Table 1, Figures 1 and 2.

**Table 1:** Survival rates at 10, 15 and 20 years of follow‐up by Kaplan–Meier curves and estimated survival rate using competing risk analysis.

Time of follow‐up (years)	*N* monitored	Accumulated events	Estimated survival rate on Kaplan–Meier (%)	95% CI		Estimated survival rate in the presence of competitive events (%)
Revision due to any reason						
10	185	16	93.5	89.7	96.0	93.90
15	132	19	91.7	87.2	94.7	92.70
20	56	20	90.8	85.7	94.1	92.30
Revision due to aseptic loosening						
10	185	1	99.0	96.7	99.9	99.60
15	132	2	99.0	95.9	99.7	99.20
20	56	3	98.0	93.3	99.4	98.80

Abbreviation: CI, confidence interval.

### Revision surgeries

From the studied cohort, 20 cases required revision surgery. In 10 cases (3.7%), the reason for the revision was infection. 80% of these infections were caused by coagulase‐negative *Staphylococcus* (seven cases of *Staphylococcus epidermidis* and one *Staphylococcus auricularis*) while two cases were caused by *S. aureus*.

In nine of those cases, a two‐stage revision was carried out with successful outcomes in seven cases and a late arthrodesis due to persistent infection in two of them. One‐stage revision was the treatment option in one of the patients with infection eradication (Table 2).

**Table 2:** Revision surgeries due to infection.

No.	Age (primary TKA)	Germ	Time to revision (years)	Details of revision	Outcome following revision
1	63	*Staphylococcus aureus*	13.3	One‐stage revision	Infection eradicated
2	67	*Staphylococcus epidermidis*	0.86	Two‐stage revision	Infection eradicated
3	79	*S. epidermidis*	0.91	Two‐stage revision	Infection eradicated
4	65	*S. epidermidis*	3.12	Two‐stage revision	Infection eradicated
5	76	*S. epidermidis*	4.9	Two‐stage revision	Infection eradicated
6	61	*S. epidermidis*	7.75	Two‐stage revision	One‐stage revision 6 years later
7	57	*S. epidermidis*	8.99	Two‐stage revision	Arthrodesis with external fixator 7 years later
8	67	*S. epidermidis*	1.74	Two‐stage revision	Extensor apparatus breakage after 13 years and reinfection leading to arthrodesis.
9	62	*S. aureus*	0.9	Two‐stage arthrodesis	Infection eradicated
10	73	*S. auricularis* (CNS)	1.94	Two‐stage revision	Infection eradicated

Abbreviations: CNS, coagulase negative *Staphylococcus*; TKA, total knee arthroplasty.

Additionally, 10 revisions were carried out due to noninfectious reasons (Table 3): three revisions (1.1%) because of aseptic loosening; three due to periprosthetic fracture (1.1%); two due to instability (0.7%) and two because of pain (0.7%), in which neither loosening nor infection could be intraoperatively demonstrated.

**Table 3:** Revision surgeries due to aseptic loosening.

No.	Age primary TKA	Diagnosis	Time to revisión (years)	Revision details
1	66	Loosening of the tibial component	11	RHK in 2T
2	71	Loosening of femoral and tibial component	7.77	RHK in 1T
3	65	Loosening of the femoral component	18.70	Femur LCCK (PS) in 1T

Abbreviations: LCCK, legacy constrained condylar knee; PS, posterior‐stabilised; RHK, rotating hinge knee; TKA, total knee arthroplasty.

In two cases, a revision of the polyethylene due to breakage was required, solving the instability referred by patients in both cases.

### Other interventions which did not require a revision of the TKA

Of the entire cohort of 263 knees, the primary TKA was associated with primary patellar prosthetization in 15 cases; only in five cases from the rest of TKA, a secondary patellar resurfacing was required, which was performed on average 4.57 years after surgery (median 2.43 and range 1.83–13.39).

Of the five cases of patellar prosthetization, one patient required revision surgery due to loosening one year later; in two cases, the patient improved after patellar resurfacing.

A case of TKA dislocation after high‐energy trauma occurred, which required closed reduction in the operating room without the need for prosthesis revision or other interventions.

In three cases, reintervention was required for periprosthetic fracture fixation, without the need to revision of any prosthetic component.

### Radiographic analysis

A total of five patients presented some radiolucent lines in the last radiography taken after 20 years of follow‐up, but no progression of the osteolysis or clinical evidence of loosening could be objectively assessed in these cases.

In the AP view, it was in all cases around the tibial component under the medial plateau: four patients had radiolucent lines in zone 1 and one patient in zones 1 and 2. On lateral views, some radiolucent lines were found on two occasions (both in zone 1): one patient who already presented osteolysis on the AP view and another who presented it exclusively on the lateral projection.

**Figure 3:**
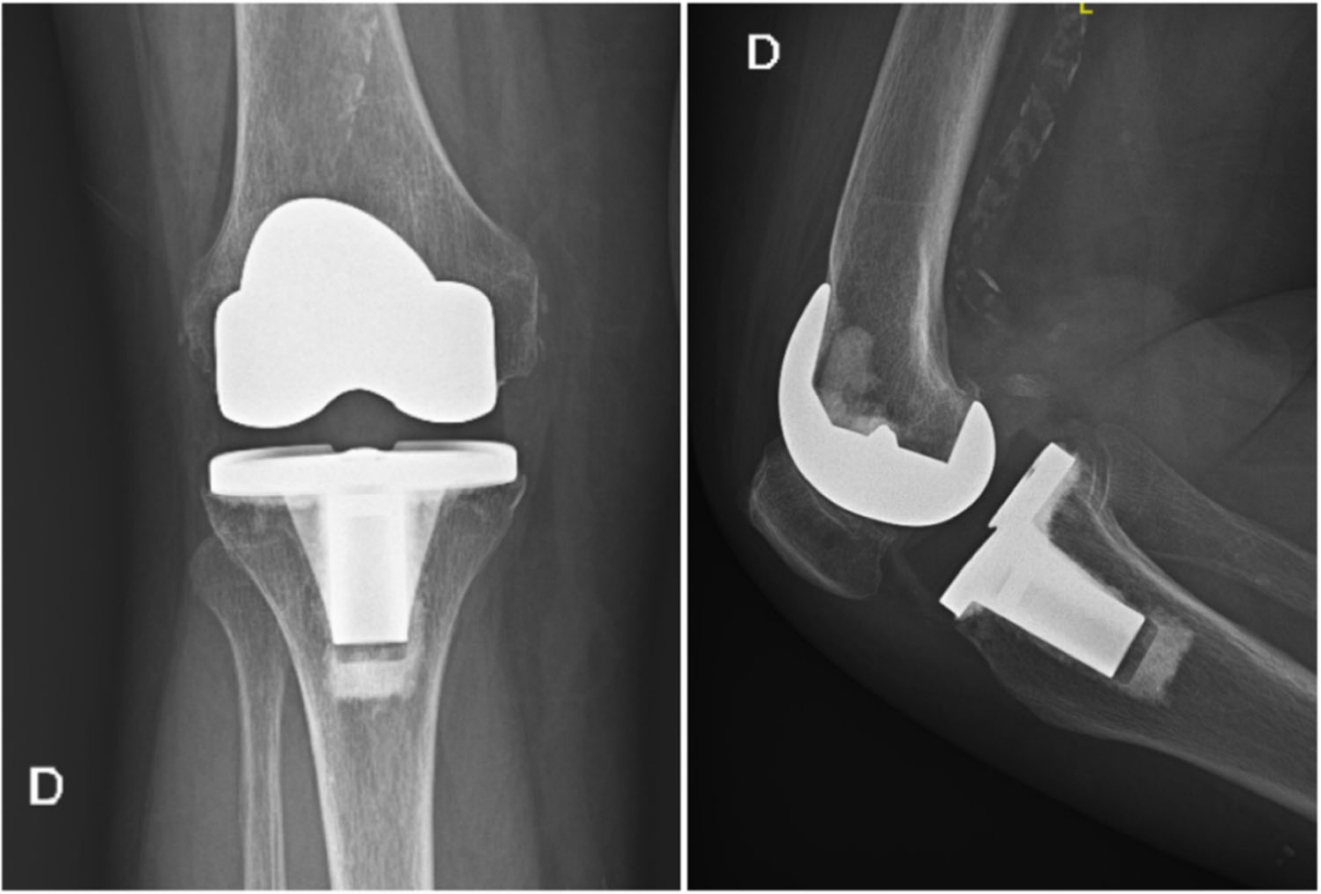
Standing anteroposterior and supine lateral X‐ray at 20.5 of follow‐up.

An example of the radiograph of a knee arthroplasty of our cohort after 20 years is shown in Figure 3.

## DISCUSSION

### Risk of revision

Long‐term studies represent one of the most important tools at our disposal for evaluating both the outcomes and the effectiveness of total joint arthroplasty surgeries. They also enable us to gain a deeper understanding of the performance of specific implants, facilitating the selection of the most suitable options for every patient. Furthermore, the conclusions drawn from such studies help us address our patients' needs and expectations when undergoing TKA procedures.

In our work, we decided to study the results for the NexGen LPS (Zimmer) prosthesis as it is one of the most widely used implants globally. This implant was developed in 1995 as an evolution of the Insall‐Burstein PS prosthesis, with the aim of improving patellofemoral biomechanics and reducing the complications associated with it [12].

In reference to this specific implant, studies have been mainly conducted with follow‐up periods of up to 15 years [3, 21, 23]. Few studies provide us with information from longer follow‐up periods. Lee et al. [19] showed survivorship of 86.4% (95% CI: 80.9%–91.9%) of TKA after 20‐year follow‐up, while Kim et al. found in our cohort a survival rate of 98% (95% CI: 93%–100%) after 20 years, with aseptic loosening being the main reason for revision [13]. These data are consistent with what we observed in our review, with an implant survivorship of 90.8% after 20 years and an estimated overall survival rate of 92.30%, with a revision rate due to aseptic loosening of 1.1%.

Our results at 20 years, as well as at 5, 10 and 15 years (reflected in Table 1) are comparable to other mid‐ to long‐term studies in the literature, despite the fact that these other studies do not differentiate the level of constraint and include cruciate retaining implants [6]. At 15 years, these studies of this implant show a survival rate of 94.7% (95% CI: 90.0%−98.5%) in 132 TKA.

Additionally, we present a low rate of loss to follow‐up from the initial cohort in comparison to the cited studies: six cases in 263 initial patients [9, 23, 24].

Regarding the most recent national registers, the latest UK National Joint Registry Annual Report from 2022 showed a higher revision for the NexGen stemmed option tibial component, which prompted a voluntary recall by Zimmer‐Biomet because the specific combination of the Stemmed Option Tibial Components with the NexGen Flex® femoral components had an increased cumulative revision risk compared to all other posterior‐stabilised TKA. Compared to all other posterior‐stabilised TKA, it showed an overall revision rate ratio of 1.73 (95% CI: 1.55%–1.92%) (*p* < 0.001) and an increased cumulative revision risk ratio for aseptic tibial loosening of 3.49 (95% CI: 2.99%–4.04%) (*p* < 0.001) [1].

In our study, we showed a lower risk of revision at 20 years, with a risk of revision lower than 2% (95% CI: 0.6%–6.7%) in contrast to the 6.75% (95% CI: 6.27%–7.27%) published in the registry for this implant at 19 years. We also found a lower rate of aseptic loosening for this implant in our cohort, being the cause of revision in only 15% of the revised TKA.

A slightly higher survival rate was observed in our study compared to the data collected by the Australian Orthopedic Association National Joint Replacement Registry 2022 Annual Report [2], which presents for the NexGen® LPS cemented prostheses a revision rate to 19 years of 8.8% (95% CI: 7.5%–10.4%) in 297 PTR, while the rate observed in our study is 7.7%.

**Figure 4:**
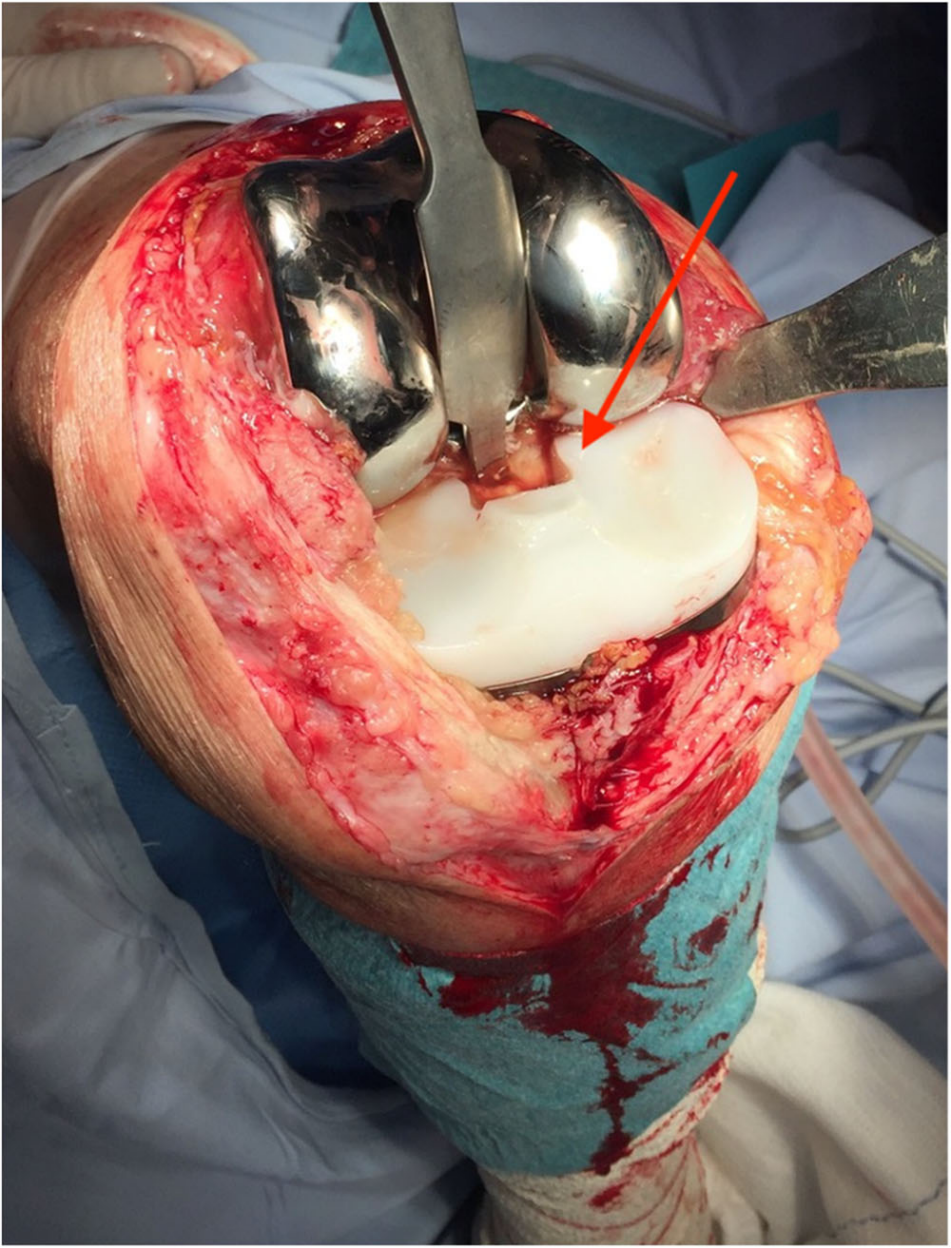
Intraoperative image of broken polyethylene tibial post (red arrow).

Regarding the two cases of polyethylene tibial postfracture (0.76%), they were resolved by replacing polyethylene. This infrequent complication, described in this and other implants, appears in less than 1% of the cases, being the same outcomes reported in the literature [5, 14, 16]. An intraoperative image can be observed in Figure 4.

In relation to the radiological assessment, in only five TKA, a radiolucent line appeared during the follow‐up but none of the cases showed any progression or ended up as loosening of the implant. This was also described in previous studies as an uncommon finding that mainly appears under the tibial component, but is not progressive or concerning in terms of increased risk of revision [8, 17].

### Patellar resurfacing

Concerning our study, the rate for primary patellar resurfacing was lower than 6%. Reviewing the existing literature, we have noted the ongoing controversy surrounding the inclusion of this surgical procedure in knee surgeries, since neither a functional improvement nor a lower rate of anterior pain has been proven, and how every study concludes this action to be carried out as each surgeon's usual practice states [10].

Additionally, only five patellar prosthetization surgeries were performed because of anterior pain after primary TKA, which contrasts the conclusions obtained by Huitema et al. [11]. In some studies, secondary patellar resurfacing due to anterior pain has been demonstrated to improve symptoms of 35% of the patients, similar results to those found in our cohort (we found improvement in two of the cases of deferred patellar resurfacing). Early revision has been shown to result in higher satisfaction rates [10].

### Analysis of competitive risk versus KM method

It has been described that KM curves can overestimate survivorship in this kind of study [4, 7, 18] and, therefore, analysis of competitive risks has been used in this work.

In studies of survivorship by KM curves, the individuals who have not experienced the primary event (implant revision) and those who are lost in follow‐up for any cause are censored. The censored individuals might therefore be at risk of revision, regardless of the reason why they were censored, so the analysis is inaccurate when there are other factors that can change the probability of the event. In a study population with advanced age, mortality rates are necessarily high when the cohort is followed for two decades. Therefore, if a subject dies during the study period prosthesis revision will not occur and this can result in an exaggerated and inaccurate survival rate.

The limitations of the study include its retrospective design, which in combination with the advanced age of the patients increases the probability of losing subjects (six TKA) as well as a long‐term follow‐up finds a high number of deceased patients (158 deceased patients in 263 TKA).

Most of these old patients suffer diseases or disabilities that prevent them from going to the clinic for physical and radiologic examination, which explains the reduced number of X‐rays or physical exams in the results. Moreover, both limitations have been affected or hindered by the SARS‐CoV2 pandemic.

## CONCLUSION

This study provides survivorship data after longer follow‐up periods, which is difficult to find in the literature studies after up to 20 years of follow‐up. The study likely provides valuable information on the effectiveness and durability of this implant over time in our cohort and showed a very low risk of revision after a long follow‐up period, especially in comparison to other studies that may only report on shorter follow‐up periods.

## AUTHOR CONTRIBUTIONS

**José Luis Patiño Contreras**: Study design; data gathering; manuscript drafting. **Victoria Sebastián Pérez**: data gathering; manuscript drafting. **Checa García Antonio**: Data interpretation; manuscript reviewing. **Montejo Sancho Jorge**: Manuscript reviewing; data interpretation. **Martínez Martín Javier**: Study design; final supervision; manuscript reviewing. All authors have contributed significantly to the study, approved the article and agreed with the submission.

## CONFLICT OF INTEREST STATEMENT

Javier Martínez Martín collaborates as a consultant for Zimmer Biomet. The remaining authors declare no conflict of interest.

## ETHICS STATEMENT

The questionnaire and methodology were approved by the local Ethics Committee of Alcorcón University Hospital. Given the methodology of this retrospective study, the ethics committee of the institution approved a waiver of the need for informed consent.

## Data Availability

The data sets used and/or analysed during the current study are available from the corresponding author upon reasonable request.
